# Cingulate protein arginine methyltransferases 1 regulates peripheral hypersensitivity *via* fragile X messenger ribonucleoprotein

**DOI:** 10.3389/fnmol.2023.1153870

**Published:** 2023-04-20

**Authors:** Cheng Wu, Hui-Fang Shang, Yong-Jie Wang, Jing-Hua Wang, Zhen-Xing Zuo, Yan-Na Lian, Li Liu, Chen Zhang, Xiang-Yao Li

**Affiliations:** ^1^Zhejiang University-University of Edinburgh Institute, Zhejiang University School of Medicine, Zhejiang University, Haining, China; ^2^Biomedical Sciences, College of Medicine and Veterinary Medicine, The University of Edinburgh, Edinburgh, United Kingdom; ^3^International Institutes of Medicine, The Forth Affiliated Hospital, Zhejiang University School of Medicine, Zhejiang University, Yiwu, China; ^4^NHC and CAMS Key Laboratory of Medical Neurobiology, MOE Frontier Science Center for Brain Research and Brain-Machine Integration, School of Brain Science and Brain Medicine, Zhejiang University, Hangzhou, China; ^5^Department of Neurosurgery, Tenth People’s Hospital, Shanghai, China; ^6^Core Facilities of the School of Medicine, Zhejiang University, Hangzhou, Zhejiang, China; ^7^School of Basic Medical Sciences, Capital Medical University, Beijing, China

**Keywords:** protein arginine methyltransferases 1, peripheral hypersensitivity, cingulate cortex, peripheral nerve injury, fragile X messenger ribonucleoprotein

## Abstract

The deficit of fragile X messenger ribonucleoprotein (FMRP) leads to intellectual disability in human and animal models, which also leads to desensitization of pain after nerve injury. Recently, it was shown that the protein arginine methyltransferases 1 (PRMT1) regulates the phase separation of FMRP. However, the role of PRMT1 in pain regulation has been less investigated. Here we showed that the downregulation of PRMT1 in the anterior cingulate cortex (ACC) contributes to the development of peripheral pain hypersensitivity. We observed that the peripheral nerve injury decreased the expression of PRMT1 in the ACC; knockdown of the PRMT1 *via* shRNA in the ACC decreased the paw withdrawal thresholds (PWTs) of naïve mice. Moreover, the deficits of FMRP abolished the effects of PRMT1 on pain sensation. Furthermore, overexpression of PRMT1 in the ACC increased the PWTs of mice with nerve injury. These observations indicate that the downregulation of cingulate PRMT1 was necessary and sufficient to develop peripheral hypersensitivity after nerve injury. Thus, we provided evidence that PRMT1 is vital in regulating peripheral pain hypersensitivity after nerve injury *via* the FMRP.

## Highlights

- Peripheral nerve injury decreased the expression of PRMT1 in the ACC.
- The downregulation of cingulate PRMT1 was sufficient to induce peripheral hypersensitivity.
- FMRP was necessary for PRMT1’s regulation of peripheral hypersensitivity.
- The downregulation of cingulate PRMT1 was necessary for peripheral hypersensitivity.

## 1. Introduction

The absence of the RNA-binding protein Fragile X messenger ribonucleoprotein (FMRP) results in fragile X syndrome. This condition causes intellectual disabilities and social deficits in affected individuals (Hagerman et al., 2018). The FMRP regulates the synaptic plasticity in multiple brain regions, including the anterior cingulate cortex (ACC) (Zhao et al., 2005) and hippocampus (Martinez and Tejada-Simon, 2018; Asiminas et al., 2019; Prieto et al., 2021). ACC and hippocampus share common molecular mechanisms regulating synaptic transmission, and ACC is a vital target for regulating neuropathic pain (Bliss et al., 2016). It has been extensively reported that ACC could be activated by neuropathic pain and enhanced excitatory synaptic transmissions (Xu et al., 2008; Bliss et al., 2016; Wang et al., 2017). Furthermore, previous studies have detected sensory abnormalities in the *Fragile X messenger ribonucleoprotein 1* (*Fmr1)* knock-out (KO) mice (Wang et al., 2016; He et al., 2017) and FXS patients (Juczewski et al., 2016), who have more robust and more frequent responses and reduced habituation to sensory stimulations, including olfactory, auditory, visual, tactile, and vestibular stimuli, as measured by electrodermal responses (Miller et al., 1999; Kazdoba et al., 2014; Kozono et al., 2020). More specifically, *Fmr1* KO mice exhibit hyporesponsiveness to inflammatory and neuropathic pain. Also, patients with FXS display altered sensory processing of nocifensive response, including sensory hyperresponsiveness and hyporesponsiveness (Wang et al., 2016). Furthermore, previous studies have detected sensory abnormalities in the *Fragile X messenger ribonucleoprotein 1* (*Fmr1)* KO mice (Wang et al., 2016; He et al., 2017) and FXS patients (Juczewski et al., 2016). Consistently, the deficit of FMRP is desensitized to pain after nerve injury (Price et al., 2007). These studies indicate that FMRP has a critical role in regulating somatic sensation.

The liquid–liquid phase separation of the FMRP-RNA complex is critical to delivering the bonded RNA to the target locations (Kim et al., 2019; Tsang et al., 2019); this process is vital for RNA transporting and initiating protein translation (Tsang et al., 2019). PRMT1 was the first identified mammalian methyltransferase (Bedford and Clarke, 2009). It catalyzes the formation of ω-NG-monomethyl arginine (MMA) and asymmetric di-methylarginine (ADMA) (McBride and Silver, 2001) and regulates ~85% of total protein arginine methylation observed in cultured rat fibroblasts and mouse liver (Blanc and Richard, 2017). Previous studies showed that the activities of PRMT1 determined the methylated level of FMRP (Stetler et al., 2006; Blackwell et al., 2010), which regulates the phase separation of the FMRP-RNA complex (Tsang et al., 2019), and determines the binding ability of FMRP (Blackwell et al., 2010). These studies indicate that PRMT1 has a critical role in regulating the functions of FMRP (Stetler et al., 2006; Blackwell et al., 2010), especially in the central nervous system (CNS). However, this point has not been evaluated. By employing the pain evaluation assay, we evaluated the involvement of PRMT1-FMRP in ACC’s pathological changes of pain. Our data suggest that PRMT1 may be a new target for chronic pain treatments in the future.

## 2. Materials and methods

### 2.1. Animals and **CPN/CFA model**

Adult (8–10 weeks old) male C57BL/6 J mice were purchased from Shanghai SLAC. *Fmr1* knock-out (*Fmr1 KO*, FVB;129P-*Fmr1*^tm1Cgr^/J) mice with an FVB genetic background were gifted from Prof. Chen Zhang (Capital Medical University, Beijing, China). Mice were bred and maintained in the experimental animal center of Zhejiang University. *Fmr1* KO mice were generated by crossing heterozygous female *Fmr1*^+/−^ mice with male *Fmr1*^+/y^ mice. The offspring were genotyped by PCR using mouse tail-tip DNA and standard primers (5′-CTTCTGGCACCT CCAGCTT-3′), WT primers (5′-TGTGATAGAATATGCAGCATGTGA-3′) for 131 bp and mutant allele-specific primers (5′-CACGAGACTAGTGAGACGTG-3′) for 400 bp. The PCR products were visualized with ethidium bromide staining. Animals were maintained on a 12 h/12 h light/dark cycle. Food and water were provided *ad libitum*. The animal care and use committee of Zhejiang University approved all mice protocols.

For the CPN ligation mouse model, in brief, mice were anesthetized with isoflurane (1% ~ 3%, as needed, RWD, Shenzhen, China, R510-22). The left CPN between the anterior and posterior groups of muscles was slowly ligated with a chromic gut suture 5–0 (Jinhuan, Shanghai, China, F503) until the digits began to twitch. The skin was sutured using a 5–0 silk suture and cleaned with povidone-iodine (Caoshanhu, Nanchang, China). Sham surgery was conducted in the same manner, but the nerve was not ligated. All animals were kept in a standard living chamber after surgery. The mice could recover for at least 7 days before the behavioral test.

To induce inflammatory pain, 10 μl of 50% CFA (Sigma, St. Louis, MO, F5881) was injected subcutaneously into the dorsal surface of one hindpaw. As a Sham group, saline was injected.

### 2.2. Mechanical allodynia test

The von Frey behavioral test was performed according to the up-down algorithm described by Chaplan et al. (1994). To determine speedy withdrawal or paw flinching evoked by mechanical stimuli, animals were placed on a raised mesh grid and covered with a clear plastic box for containment. Calibrated von Frey filaments were applied to the middle of the plantar surface of each paw until the filament bent. Brisk withdrawal or paw flinching was considered a positive response. Lifting of the paw due to normal locomotor behavior was ignored. In the absence of a response, the filament of the following greater force was applied. Following a response, the filament of the next lower force was applied. The tactile stimulus producing a 50% likelihood of a withdrawal response was calculated and treated as the paw withdrawal threshold (PWT).

### 2.3. Cannulation and microinjection

For the cannula surgery and microinjection, mice were anesthetized with isoflurane (1–3%, as needed, RWD, Shenzhen, China, R510-22) inhalation of 100% oxygen with a flow of 0.5 L/min delivered by facemask. The scalp was shaved and then cleaned with iodine (Caoshanhu, Nanchang, China) and alcohol. The head of each mouse was fixed into a stereotaxic adapter mounted on a stereotaxic frame (RWD, Shenzhen, China, 68,025), and eye ointment (Cisen, Jining, China) was applied to the eyes. An incision was made over the skull, and the surface was exposed. Two small holes were drilled above the ACC, and the dura was gently reflected. Guide cannulas were placed 0.7 mm anterior to the bregma, 0.3 mm lateral to the midline, and 1.75 mm ventral to the surface of the skull. For the microinjection, the mice were restrained in a plastic cone (RWD, Shenzhen, China, 68,025), and a small hole was cut in the plastic overlying the microinjection guides. The dummy cannulas were removed, and the microinjection cannula was inserted into the guide. A 30-gage injection cannula was placed 0.7 mm lower than the guide. AMI-1 (0.5 μL, 0.1 ng/μL, Sigma-Aldrich, Saint Louis, MO 63103, United States, A9232) was bilaterally delivered at 0.5 μL/min using a syringe driven by an infusion pump (Alcott, Shanghai, China, ALC-IP600). The volume delivered was confirmed by watching the movement of the meniscus down a length of calibrated polyethylene (PE10) tubing. After delivery to each side of the brain, the injection cannula was left in place for 1 min to prevent any solution from flowing back up the guide. The cannula was then retracted and inserted into the opposite side of the brain. Ten min after microinjection, the mechanical allodynia test was administered.

### 2.4. Constructs, viral packaging, and stereotactic injection

The adeno-associated viruse 8 (AAV8) was constructed for overexpressing FMRP (NM_008031.3) and PRMT1 (NM_019830.3). The lentiviruses were constructed for the knockdown of FMRP (5’-GCTGTTGGTGGTTAGCTAAAG-3′) and PRMT1 (5′-GCATTAAAGACGTGGCCATCA-3′). All the viruses were packaged by OBiO Technology (Shanghai) Corp., Ltd. (Shanghai, China). For viral injection, mice were anesthetized with ketamine (100 mg/kg of body weight) and xylazine (8 mg/kg) by intraperitoneal (IP) injection and placed in a stereotactic frame. Mice were injected bilaterally with purified and concentrated AAV8 or lentivirus into the ACC (1 μl, coordinates from Bregma: −0.7 mm anterior/posterior, ±0.3 mm medial/lateral, −1.75 mm dorsal/ventral) using glass microelectrodes at a slow rate (100 nL/min). The injection microelectrode was slowly withdrawn 10 min after the virus infusion. Experiments were started from at least 21 days for AAV and 10 days for lentivirus after the injection. The injection sites were examined at the end of all the behavioral tests, and only data from animals with correct injections were included. Brain slices of ACC were directly examined under a fluorescent microscope. The PWT was examined in a blind manner.

### 2.5. Real-time quantitative fluorescence PCR experiment

Primers were designed by referring to the sequences of *β-actin* and *Prmt1* in GenBank. The specific sequence information is shown in Table 1 below. Hangzhou Youkang Biological Co., LTD synthesized the designed primers.

**Table 1:** Primer sequences for real-time quantitative fluorescence PCR.

Primer	Primer suquence(5′-3′)
*Prmt1 forward*	CTACTTTGACTCCTATGCCCACT
*Prmt1 reverse*	TGTCTTTGAAGAGATGCCGAT
*β-actin forward*	TGTTACCAACTGGGACGA
*β-actin reverse*	GTCTCAAACATGATCTGGGTC

The mice were lightly anesthetized with isoflurane and then decapitated. ACC was dissected after 7 days of CPN ligation. According to the TransZol Up Plus RNA kit (TransGen Biotech, Beijing, China, ER501-01), RNA was extracted from the ACC, and its purity and concentration were measured. A total RNA of 1.0 μg was taken from each group, and reverse transcription was performed by using the reverse transcription reagent HiScript^®^II Q RT SuperMix (Vazyme, Nanjing, China, R223). The transcribed cDNA was used as the real-time quantitative PCR reaction template, as shown in Table 2. The real-time PCR experiment obtained the Ct value, amplification curve, and dissolution curve. According to the formula ΔCt = CT_Target_ -CT_actin_, the average Ct value of 3 duplicates of each sample was taken to calculate the ΔCt value of each target gene relative to the reference gene *β-actin*, using 2^-ΔΔCt^ (Livak and Schmittgen, 2001) method, the relative expression level of each gene was calculated.

**Table 2:** Real-time quantitative PCR reaction system and procedure.

Reaction system	Volume (μL)	Program
2 × PCR mix	5	95°C 3 min
Forward primer	0.4	95°C 5 s
Reverse primer	0.4	40 cycles
cDNA	1	60°C 15 s
H_2_O	3.2	

### 2.6. Western blot analysis

The mice were lightly anesthetized with isoflurane and then decapitated. The regions needed were dissected after 7 days of CPN ligation and then homogenized in a RIPA buffer (50 mM pH 7.6 Tris-Cl, 150 mM NaCl, 1 mM EDTA, 1% NP-40, 0.1% SDS, 1 mM DTT, 0.5% sodium deoxycholate) containing a protease inhibitor cocktail. After centrifugation, the supernatants were used for protein quantification by the Bradford assay. Electrophoresis of equal amounts of total protein was performed on SDS-polyacrylamide gels. The separated proteins were transferred onto polyvinylidene membranes at 4°C. The membranes were blocked for 2 h with 5% milk in TBST (Tris-buffered saline with Tween-20, room temperature) and incubated with a primary antibody (PRMT1, 1:1000, Abcam, Cambridge, UK, ab73246; PRMT1, 1:1000, Proteintech, Wuhan, China, 11,279-1-AP) at 4°C for overnight. After being washed, the membranes were incubated for 1 h with the appropriate HRP-coupled secondary antibody (Jackson immune research, goat anti-rabbit, 111–035-144) diluted 1:3000, followed by enhanced chemiluminescence detection of the proteins with Western Lightning Chemiluminescence Reagent Plus (Life Technologies, California, United States, 1863097), according to the manufacturer’s instructions. To verify equal loading, we probed the membranes with antibodies against tubulin (1:10000, Sigma, T5201) or actin (1:5000, Sigma, A3853). The density of the immunoblots was measured with the NIH ImageJ program.

### 2.7. Immunostaining

Mice were anesthetized with 1% pentobarbital sodium (50 mg/kg, Sigma-Aldrich, Saint Louis, MO 63103, United States, P3761) and subjected to cardiac perfusion with 0.01 M phosphate-buffered saline (PBS) followed by 4% paraformaldehyde (PFA) in PBS. Brains were post-fixed overnight in PFA at 4°C, then transferred to 15% sucrose in PBS, followed by 30% sucrose until saturated. The brain was embedded in Tissue-Tek O.C.T. compound, frozen in liquid nitrogen, and stored at-80°C before being sliced to 25 μm coronal cryostat sections at-20°C (Leica CM3050S). Free-floating sections containing ACC were washed with PBS. For immunostaining, sections were incubated with blocking buffer (5% normal goat serum and 0.3% Triton X-100 in PBS) for 1 h at room temperature and incubated with primary antibodies (FMRP, 1:150, Proteintech, Wuhan, China, 66,548-1-Ig; PRMT1, 1:150, Abcam, Cambridge, UK, ab73246) overnight at 4°C. Sections were washed in PBS and then added appropriate secondary antibodies (goat anti-mouse 594, Life Technologies, California, United States, A-11005; goat anti-rabbit 488, Life Technologies, California, United States, A-11034) for 2 h at room temperature. Sections were rewashed for 3 × 10 min in PBS. Following washing, the stained coverslips were mounted using Fluoroshield Mounting Medium with DAPI (Abcam, Cambridge, UK, ab104139) for image collection.

### 2.8. Open-field test

White-colored plastic boxes were used as the open field chambers (dimension: 45 × 45 × 45 cm^3^). Mice were individually placed into the center of the chambers and allowed to explore for 5 min freely. The locomotion and exploratory behaviors of mice were recorded with Anymaze software (Stoelting, UK). The total traveling distance was used to evaluate locomotor activity.

### 2.9. Cell line transfection

Human embryonic kidney cells (HEK-293 T) were cultured in DMEM (Corning Cellgro, Virginia, United States, 10-017-CVA) containing 5% fetal bovine serum (Excel. Bio, Hefei, China, FCS500). The medium also contained 5% F12 (Gibco™, California, USA, 11765–054) and penicillin/streptomycin (Gibco™, California, USA, 15140122). 0.25% trypsin (Gibco™, California, USA, 25200056) digestion was performed every 2 ~ 3 days. All the cultures were placed in a 3.5 cm NEST dish at 37°C and 5.0% CO_2_. FMRP-GFP plasmid was transfected into HEK-293 T using Lipofectamine™ 3,000 transfection reagent (Invitrogen™, California, United States, L3000075). After transfection for 12–14 h, the fluorescence of the fluorescent protein could be seen under a fluorescence microscope (Olympus, Tokyo, Japan, CKX53). AMI-1 (Sigma-Aldrich, Saint Louis, United States, A9232) was added to the medium at the final concentration of 100 μM after transfection for 24 h and incubated for 1 h continuously.

### 2.10. Culturing hippocampal neurons and transfection

Kill three or four postnatal rat pups using an approved method of euthanasia; the head was cut off quickly, the hippocampus was taken out, and the tissue was chopped up on an ice plate and put into 1 ml 0.25% trypsin for digestion at 37°C for 12 min. After complete digestion, the tissue was blown 12 times with a rounded tip three times to obtain the supernatant. Centrifugation followed, in which the cell suspension and debris were stratified, and the cell suspension was carefully sucked out. Plate 1 × 10^6^ cells per 3.5 cm dish. Change half of the culture medium with fresh medium, including Neurobasal Medium (Invitrogen™, California, United States, 21103–049), GlutaMax-1 (Invitrogen™, California, USA, 35050–061), and B27 supplement (Invitrogen™, California, United States, 17504–044), every 3 days. Ten days after plating, neurons were transfected with AAV8 carried with GFP or *Fmr1*. 48 h after transfection, AMI-1 was added to the medium at the final concentration of 100 μM and incubated for 1 h continuously.

### 2.11. Quantification of cells with FMRP granule clusters

Cells were initially fixed with 4% PFA for 10 min for cluster quantification after AMI-1 incubation. DAPI was used for nuclear counter-staining. Images were taken using Olympus IX83-FV3000. To quantify the percentage of cells with FMRP granule clusters, more than 50 cells were counted for each sample.

### 2.12. Data analysis

GraphPad 8.0 was used to plot and fit the data. Statistical comparisons were made using the paired or unpaired *t*-test, one-way or two-way ANOVA (Student–Newman–Keuls test was used for *post hoc* comparison), and chi-square test. All data were presented as the mean ± SEM. In all cases, *p* < 0.05 is considered statistically significant.

## Results

### Peripheral nerve injury decreased the expression of PRMT1 in the anterior cingulate cortex (ACC)

**Figure 1:**
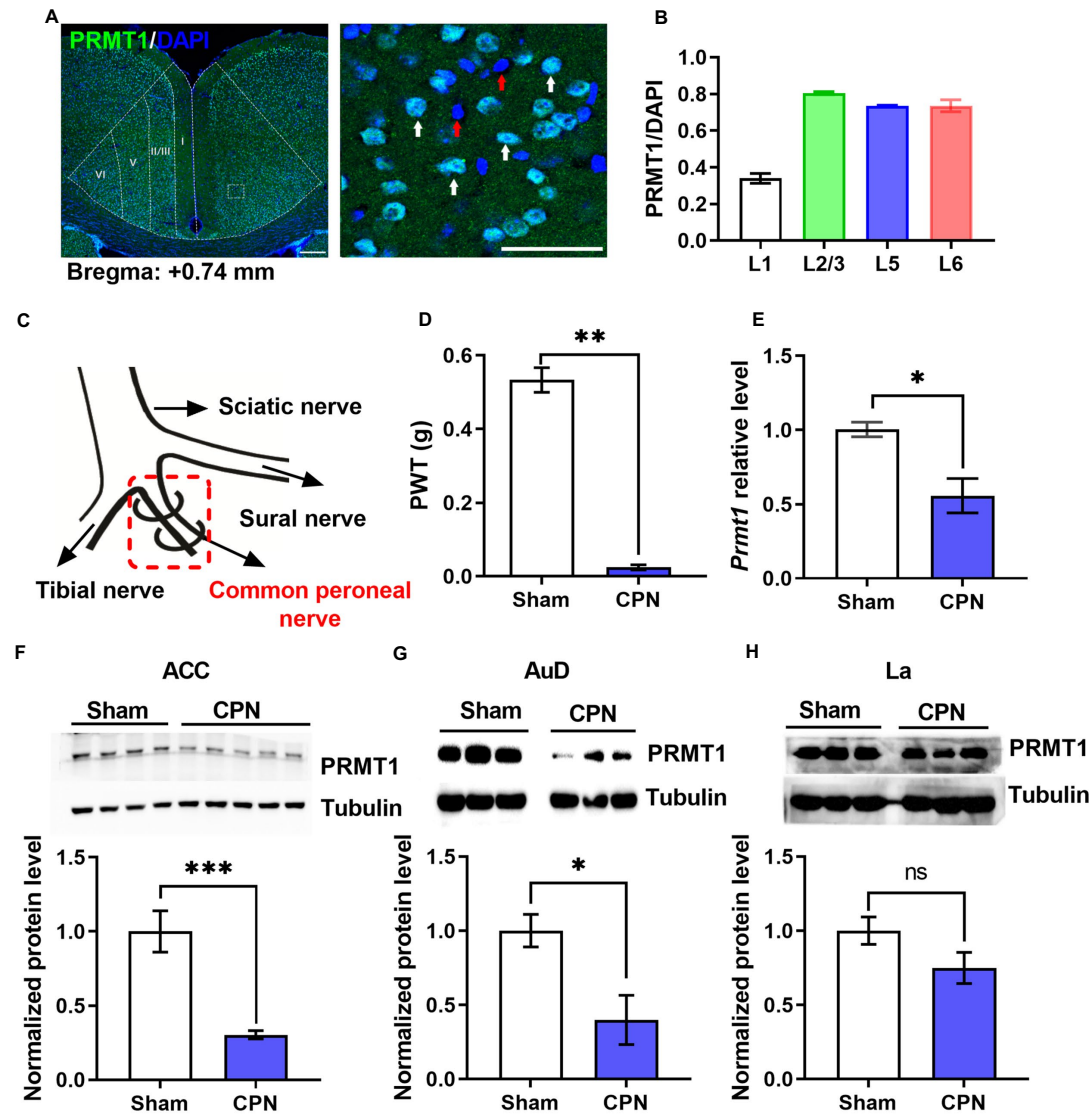
Peripheral nerve injury decreased PRMT1. **(A)** Left: representative photomicrograph of PRMT1 (green) in the anterior cingulate cortex (ACC). Right: zoomed-in view of the boxed region in the left panel. Blue: DAPI; green: PRMT1; red arrow: DAPI; white arrow: PRMT1 and DAPI; scale bars, 200 μm (left), 50 μm (right). **(B)** Quantification of PRMT1 positive cells in different layers of the ACC. **(C)** Schematic diagram of the ligation of the common peroneal nerve (CPN) in mouse. **(D)** Paw withdrawal thresholds (PWTs) assessed by von Frey behavioral test after common peroneal nerve (CPN) ligation (Sham and CPN, *n* = 6/group, unpaired *t*-test, *p* < 0.01; **p < 0.01). **(E)** *Prmt1* mRNA level in the ACC of Sham and CPN mice (Sham: *n* = 3, CPN: *n* = 3; unpaired *t-*test, *p* = 0.0237; *p < 0.05). **(F)** PRMT1 protein level in the ACC of Sham and CPN mice. Top: representative immunoblot images; bottom: statistical analysis (Sham: *n* = 4, CPN: *n* = 5; unpaired *t-*test, *p* = 0.0009; ***p < 0.001). **(G)** PRMT1 protein level in the auditory cortex (AuD) of Sham and CPN mice. Top: representative immunoblot images; bottom: statistical analysis (Sham: *n* = 3, CPN: *n* = 3; unpaired *t-*test, *p* = 0.039; *p < 0.01). **(H)** PRMT1 protein level in the lateral amygdaloid nucleus (La) of Sham and CPN mice. Top: representative immunoblot images; bottom: statistical analysis (Sham: *n* = 3, CPN: *n* = 3; unpaired *t-*test, *p* = 0.1453). Data are represented as mean ± SEM; ns, not significant.

To investigate the involvement of PRMT1 in the development of peripheral pain hypersensitivity, we first examined the expression of PRMT1 in the ACC and found that PRMT1, respectively, expressed in layer 1 (PRMT1/DAPI%; 33.95%), layer 2/3 (80.4%), layer 5 (73.41%) and layer 6 (73.55%) (Figures 1A,B). After that, we performed ligation of the common peroneal nerve (CPN) (Vadakkan et al., 2005) on naïve mice (Figure 1C), which decreased paw withdrawal thresholds (PWTs) on day seven after the operation (Figure 1D). Interestingly, the lower levels of *Prmt1* mRNA and PRMT1 protein were, respectively, observed in the ACC (Figures 1E,F). In order to examine whether the down-regulation of PRMT1 selectively occurred in ACC after CPN ligation, we also detected the PRMT1 expression in the auditory cortex (AuD) and lateral amygdaloid nucleus (La). The down-regulation of PRMT1 was also observed in the AuD but not in the La (Figures 1G,H). Cumulatively, these results demonstrate that PRMT1 in the ACC is downregulated after peripheral nerve injury.

### 2.13. Downregulation of cingulate PRMT1 was sufficient for the development of peripheral somatic hypersensitivity

**Figure 2:**
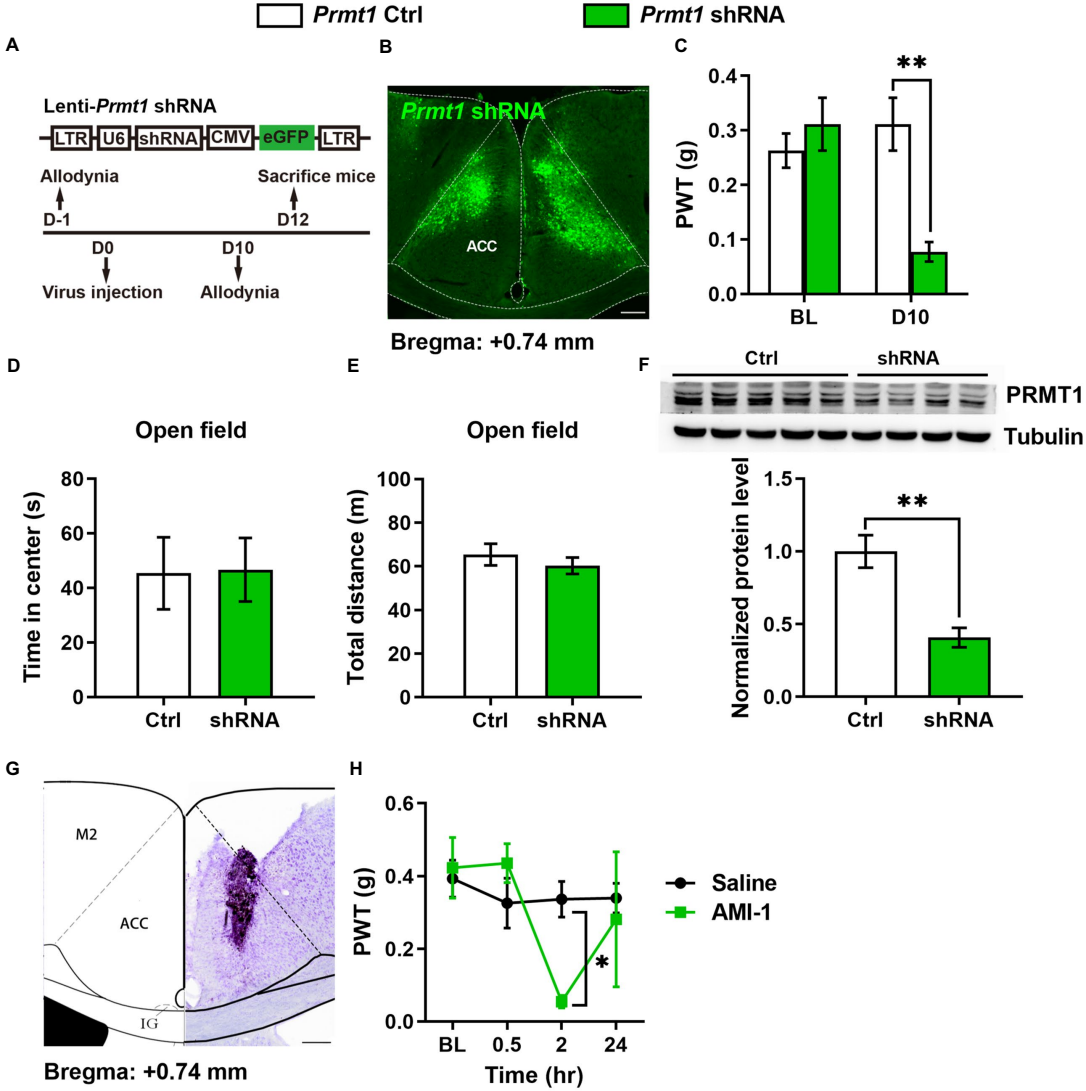
Downregulation of cingulate PRMT1 was sufficient and necessary for the development of peripheral hypersensitivity **(A)** Up: schematics of lentivirus vector engineered shRNA to knock down *Prmt1* and eGFP control (Ctrl). Bottom: the experimental paradigm for behavioral testing. scale bar, 200 μm. **(B)** Illustration of bilateral expression of lenti-*Prmt1* in C57BL/6 J mouse ACC. **(C)** PWT tests after *Prmt1* shRNA expressed in the ACC (two-way RM ANOVA, interaction: *F_(2;36)_* = 23.95, *p* < 0.01, Time: *F_(2;36)_* = 16.95, *p* < 0.01, Ctrl vs. shRNA: *F_(1;36)_* = 152.63, *p* < 0.01, Ctrl: *n* = 10, shRNA: *n* = 10; “**” indicates *p* < 0.01; BL: baseline). **(D)** Statistics for the center time in the open-field test (Ctrl: *n* = 5, shRNA: *n* = 4, unpaired *t*-test, *p* > 0.05). **(E)** Statistics for the total distance in the open-field test (Ctrl: *n* = 5, shRNA: *n* = 4, unpaired *t*-test, *p* > 0.05). **(F)** Western blots and quantification showing efficient knockdown of PRMT1 by shRNA in the ACC (Ctrl: *n* = 5, shRNA: *n* = 4, unpaired *t-*test, *p* = 0.0039; “**” indicates *p* < 0.01). **(G)** Example injection site of cannula in the ACC of C57BL/6 J mice. **(H)** PWT tests after micro infusion of PRMT1 inhibitor, AMI-1, into ACC (Two-way RM ANOVA, interaction: F*_(1;9)_* = 10.19, *p* < 0.05, Time: F*_(1;9)_* = 13.51, *p* < 0.05, saline vs. AMI-1: **(F)** *_(1;9)_* = 7.24, *p* < 0.05, Ctrl: *n* = 6, AMI-1: *n* = 5; “*” indicates *p* < 0.05). Data are represented as mean ± SEM; ns, not significant.

We next want to know whether the downregulation of cingulate PRMT1 contributes to peripheral hypersensitivity after a nerve injury. In order to answer this question, we first examined the causality of the PRMT1 downregulation, expressing the *Prmt1* shRNA in the ACC (Figures 2A,B), to peripheral pain hypersensitivity. Behaviorally, we observed significantly lower PWTs (Figure 2C) after shRNA expression but not the control (Ctrl) virus. The PRMT1 downregulation did not affect the motor function because there were no changes in the center time (Figure 2D) and travel distance (Figure 2E) in the open field. Further examinations confirmed the downregulation of PRMT1 (Figure 2F).

We also evaluated the effects of blocking the activity of PRMT1 with AMI-1, a selective and potent PRMT1 inhibitor. When we micro-infused AMI-1 into the ACC of naïve mice (Figure 2G), the PWTs markedly decreased (Figure 2H). Thus, by combining genetic and pharmacological approaches, we showed that the downregulation of cingulate PRMT1 is sufficient to induce peripheral hypersensitivity.

### 2.14. PRMT1 regulates the condensation of FMRP *in vitro*

**Figure 3:**
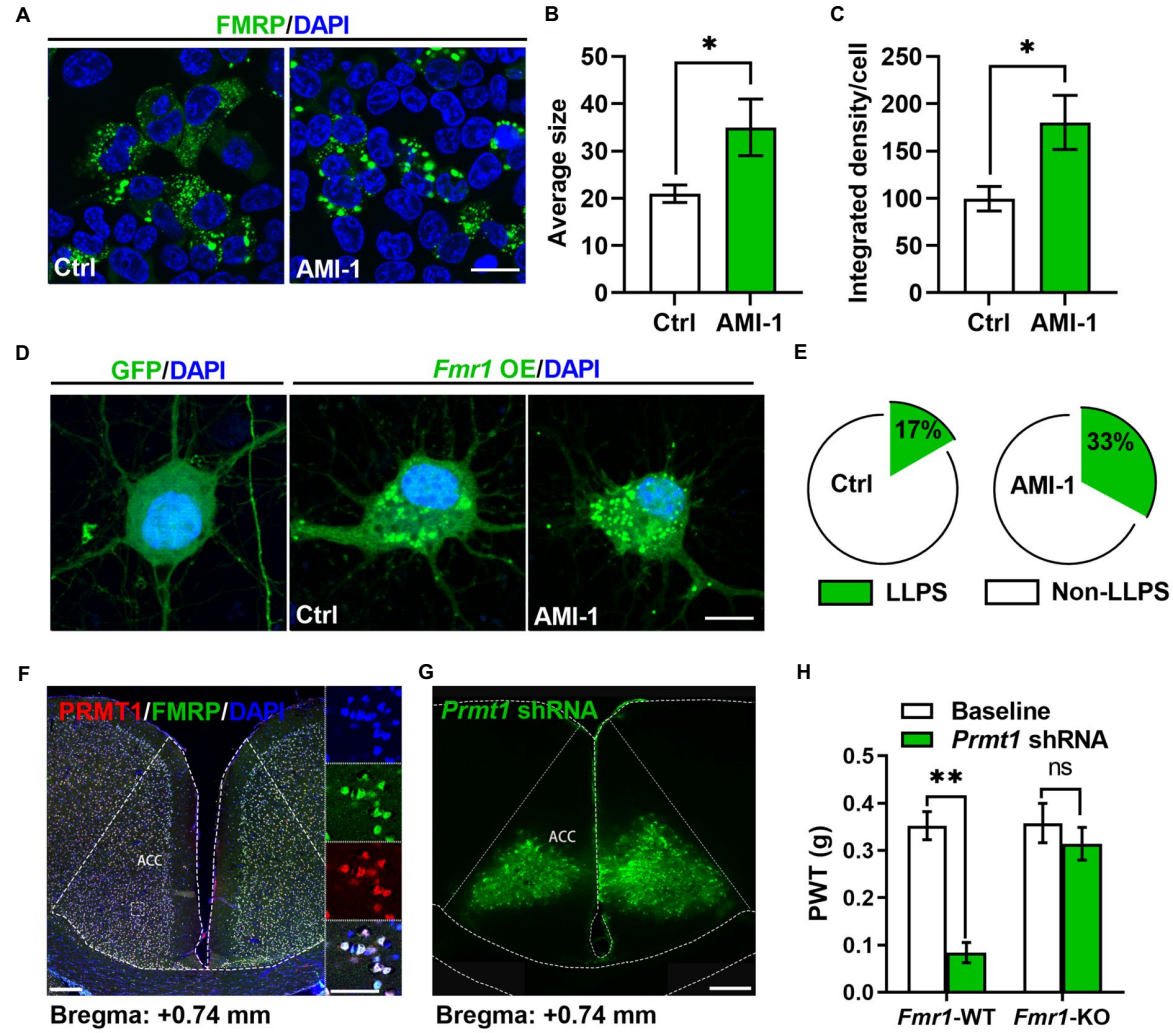
FMRP was involved in the development of peripheral hypersensitivity induced by PRMT1 downregulation. **(A)** Representative examples of control (Ctrl, left) and the application of AMI-1 (right) of FMRP-GFP in HEK-293 T cells. Scale bar, 20 μm. **(B)** The average size of the condensed FMRP-GFP clusters in Ctrl and AMI-1 groups (Ctrl: *n* = 8, AMI-1: *n* = 7; unpaired *t*-test, *p* = 0.0338; *p < 0.05). **(C)** The integrated density of the condensed FMRP-GFP clusters in Ctrl and AMI-1 groups (Ctrl: *n* = 8, AMI-1: *n* = 7; unpaired *t*-test, *p* = 0.0185; *p < 0.05). **(D)** Representative examples of GFP (left), Ctrl (middle, transfected FMRP-GFP) and the application of AMI-1 (right, transfected FMRP-GFP) of FMRP-GFP in the cultured neurons. Scale bar, 10 μm. **(E)** The percentage of condensed FMRP in the FMRP-GFP clusters after applying solvent (Ctrl group, left) or AMI-1 (right). LLPS: liquid-liquid phase separation; Non-LLPS: no liquid-liquid phase separation (Chi-square test, Ctrl vs. AMI-1, *p < 0.05). **(F)** Confocal images of FMRP-and PRMT1-expressing cells in ACC; zoomed-in view of the boxed region in the left panel (blue, DAPI; red, PRMT1; green, FMRP; scale bars, 200 μm (left), 50 μm (right)). **(G)** Illustration of bilateral expression of lenti-*Prmt1* shRNA in ACC of *Fmr1* wild-type (WT)/knock-out (KO) mice (scale bar, 200 μm). (H) PWT tests after *Prmt1* shRNA expressed in ACC of *Fmr1* WT and KO mice (Two-way RM ANOVA, interaction: *F_(2;32)_* = 10.34, *p* < 0.01, Time: *F_(2;32)_* = 14.15, *p* < 0.01, WT vs. KO: *F_(1;32)_* = 47.84, *p* < 0.01; Baseline: WT vs. KO, *p* > 0.05; WT: *n* = 8, KO: *n* = 10; **p < 0.01). Data are represented as mean ± SEM; ns, not significant.

Since the phase separation of FMRP is essential to its combining with RNA, which is regulated by PRMT1 (Tsang et al., 2019), we confirmed the regulation of PRMT1 on the phase separation of FMRP. We expressed the FMRP conjected with GFP in the HEK-293 T cell and observed cluster-like fluorescence signals in the FMRP-GFP group 24 h later. We then applied AMI-1 to inhibit the activities of PRMT1 (Figure 3A) and observed a larger size (Figure 3B) and higher fluorescence intensity (Figure 3C) of FMRP in the AMI-1 group than in the Ctrl group. This result suggests that PRMT1 regulates the condensation of FMRP in the HEK-293 T system.

We further expressed the FMRP-conjected GFP in the cultured neurons *via* the AAV virus. Consistently, we observed both dispersed and condensed GFP signals after virus transfection (Figure 3D), while, in the Ctrl group, we only observed dispersed GFP signals (Figure 3D). We then applied AMI-1 to inhibit the activities of PRMT1. Unlike the observations from HEK-293 T, we did not observe any differences in the average size and fluorescence intensity of FMRP between the two groups. However, a higher proportion of neurons had condensed FMRP (Figure 3E). Therefore, inhibiting the activities of PRMT1 in the cultured neurons also increased the condensation of FMRP, which mirrors that the activities of PRMT1 determined the methylated level of the FMRP-RNA complex.

### 2.15. FMRP was involved in the development of peripheral hypersensitivity induced by PRMT1 downregulation

Since PRMT1 regulates the liquid–liquid phase separation of the FMRP-RNA complex, this observation prompts us to explore further how PRMT1 regulates peripheral hypersensitivity and raises an interesting question of whether the FMRP is involved in regulating peripheral hypersensitivity induced by PRMT1 downregulation. Firstly, we investigated whether PRMT1 has the same distribution patterns as FMRP in the ACC. As shown in Figure 3F, about 90% of cells expressed both PRMT1 and FMRP. Next, we wanted to know whether the PRMT1-FMRP was involved in the development of peripheral hypersensitivity. When we delivered a *Prmt1* shRNA into the ACC of both *Fmr1* wild-type (WT) and knock-out (KO) mice (Figure 3G), we recorded decreased PWTs in the WT but not in the KO mice (Figure 3H). These data suggest that FMRP is critical for the development of hypersensitivity induced by PRMT1 downregulation.

**Figure 4:**
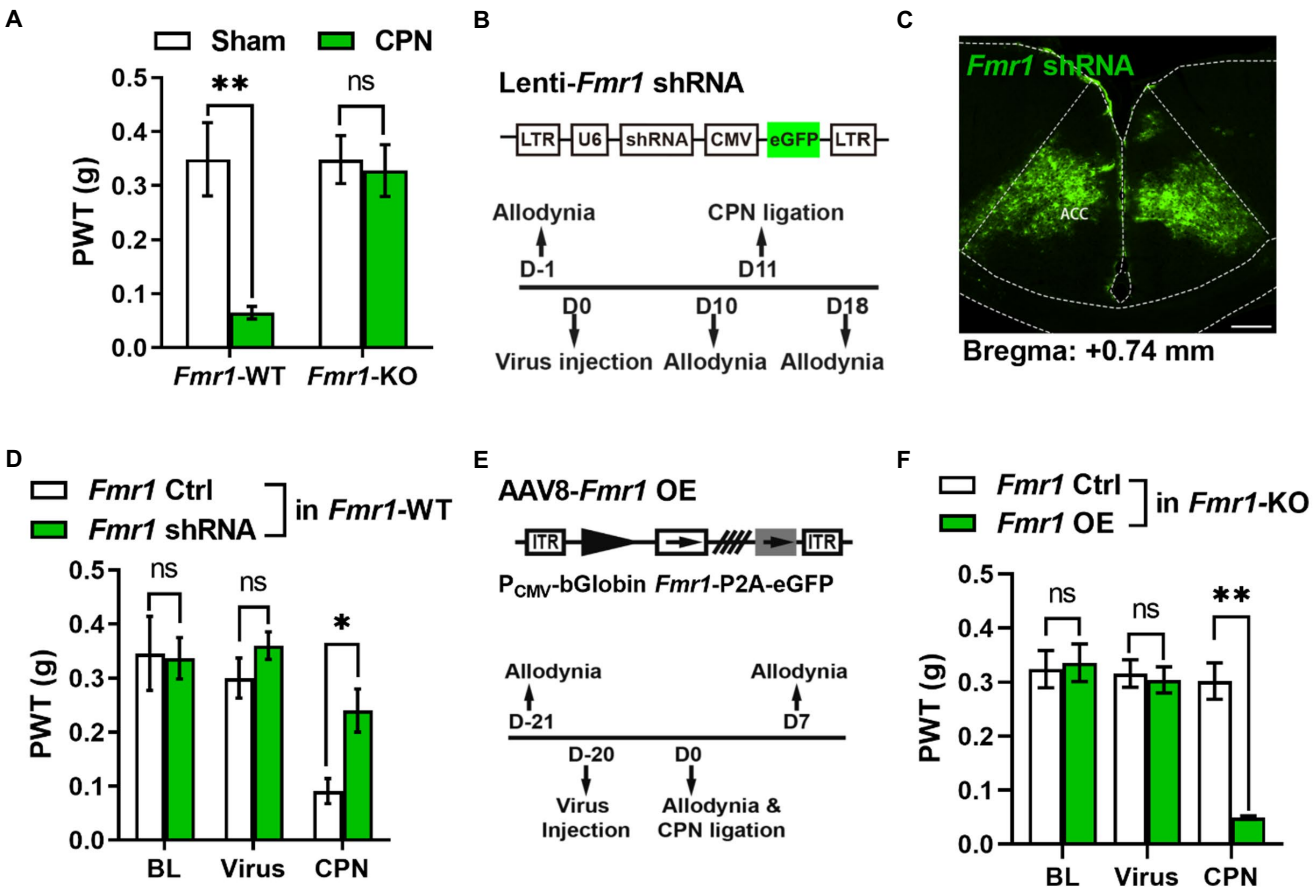
Cingulate FMRP is necessary for the development of peripheral hypersensitivity after nerve injury **(A)** PWT tests after CPN ligation in *Fmr1* WT and KO mice (Two-way RM ANOVA, interaction: *F_(1;11)_* = 4.90, *p* < 0.05, Time: *F_(1;11)_* = 6.50, *p* < 0.05, WT vs. KO: *F_(1;11)_* = 10.37, *p* < 0.01; Sham: WT vs. KO, *p* > 0.05; WT: *n* = 8, KO: *n* = 5; “**” indicates *p* < 0.01). **(B)** Upper schematics of lentivirus vectors engineered shRNA to knock down *Fmr1* and eGFP Ctrl. Lower experimental paradigm for behavioral testing after injection of *Fmr1*-shRNA virus into ACC. **(C)** Illustration of bilateral expression of lenti-*Fmr1* shRNA virus in ACC of *Fmr1* WT mice (scale bar, 200 μm). **(D)** PWT tests in *Fmr1* WT mice conducted at BL, after virus injection of either *Fmr1* Ctrl or shRNA in ACC, further after CPN ligation (Two-way RM ANOVA, interaction: *F_(3;30)_* = 1.43, *p* > 0.05, Time: *F_(3;30)_* = 19.10, *p* < 0.01, Ctrl vs. shRNA: *F_(1;30)_* = 8.24, *p* < 0.05, *n* = 6/group; “*” indicates *p* < 0.05). **(E)** Upper schematics of AAV8 vector engineered to overexpress *Fmr1* and an eGFP Ctrl. Lower experimental paradigm for behavioral testing. **(F)** PWTs in *Fmr1* KO mice detected at BL, after virus injection of either *Fmr1* Ctrl or OE in ACC, further after CPN ligation (Two-way RM ANOVA, interaction: *F_(4;72)_* = 12.36, *p* < 0.01, Time: *F_(4;72)_* = 16.11, *p* < 0.01, Ctrl vs. OE: *F_(1;72)_* = 102.31, *p* < 0.01, *n* = 10/group; “**” indicates *p* < 0.01). Data are represented as mean ± SEM; ns, not significant.

Since deficits of FMRP is extensive, the absence of FMRP in other brain areas may mask the effects of *Prmt1* shRNA on PWTs. If this is true, manipulation of the expression of cingulate FMRP will not affect the development of peripheral hypersensitivity in the *Fmr1* KO mice. We performed CPN ligation on *Fmr1* WT and KO mice to test the above deduction. We recorded lower PWTs in the WT but not in the KO mice (Figure 4A). Next, we designed an *Fmr1*-specific shRNA (Figure 4B) and delivered it to the ACC of naïve mice using lentivirus (Figure 4C). There were not any noticeable changes in PWTs on day five after virus injection (Figure 4D). Moreover, CPN ligation decreased the PWTs in mice with *Fmr1* Ctrl virus, but not in the mice expressing *Fmr1* shRNA (Figure 4D). Above-mentioned results suggest that knockdown cingulate FMRP prevents the development of hypersensitivity after nerve injury.

We next re-expressed FMRP in the ACC of *Fmr1* KO mice *via Fmr1* overexpression (OE) virus (AAV8-P_CMV_-bGlobin-*Fmr1*-P2A-eGFP) (Figure 4E) and did not find any pronounced differences in PWTs between the *Fmr1* Ctrl and OE virus groups (Figure 4F). Interestingly, in *Fmr1* KO mice, compared with the *Fmr1* Ctrl virus group, further CPN ligation significantly decreased the PWTs in *Fmr1* OE group (Figure 4F). Therefore, the functions of cingulate FMRP in the regulation of hypersensitivity were not masked by the absence of FMRP in other areas. Together, these results reveal that FMRP is involved in pain hypersensitivity after the PRMT1 downregulation.

### 2.16. PRMT1 downregulation in the ACC is necessary for developing peripheral hypersensitivity

**Figure 5:**
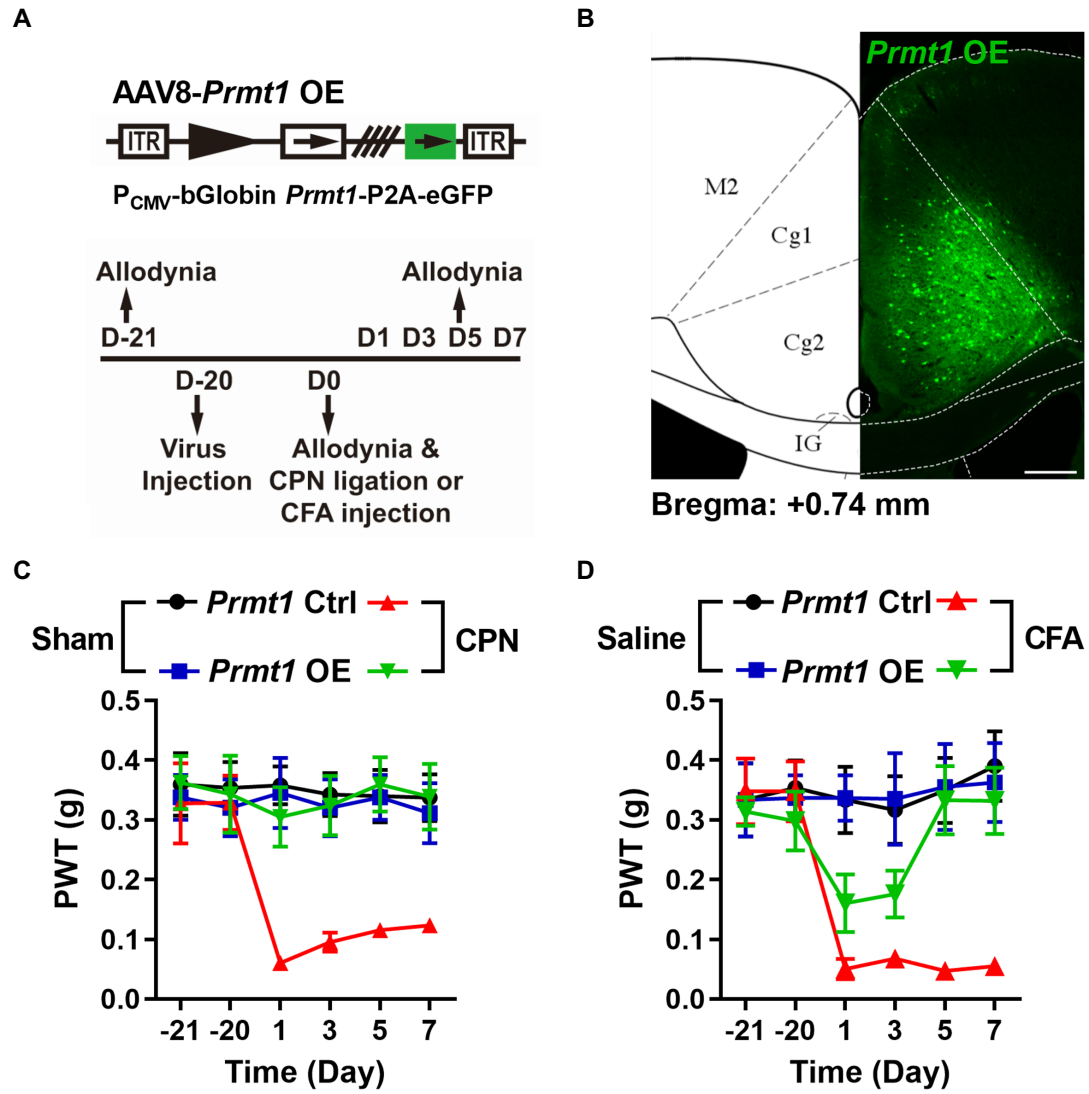
Downregulation of cingulate PRMT1 is necessary to develop peripheral hypersensitivity. **(A)** Upper schematics of AAV8 vectors engineered to overexpress PRMT1 and an eGFP Ctrl. Lower experimental paradigm for behavioral testing. **(B)** Illustration of bilateral AAV8-*Prmt1* OE virus in the mouse ACC. **(C)** PWT tests conducted in both sham and CPN-ligated mice injected either *Prmt1* Ctrl or OE virus in ACC (Sham *Prmt1* Ctrl, *n* = 9; Sham *Prmt1* OE, *n* = 8; CPN *Prmt1* Ctrl, *n* = 9; CPN *Prmt1* OE, *n* = 8, Two-way RM ANOVA, interaction: *F_(15;150)_* = 2.0, *p* < 0.05, Time: *F_(5;150)_* = 2.70, *p* < 0.05, *Prmt1* Ctrl vs. *Prmt1* OE: *F_(3;150)_* = 15.60, *p* < 0.01). **(D)** PWTs measured in both saline-and CFA-injected mice either with *Prmt1* Ctrl or OE virus in ACC (Saline *Prmt1* Ctrl, n = 6; Saline *Prmt1* OE, *n* = 6; CFA *Prmt1* Ctrl, *n* = 8; CFA *Prmt1* OE, *n* = 10, Two-way RM ANOVA, interaction: *F_(15;130)_* = 3.40, *p* < 0.01, Time: *F_(5;130)_* = 4.72, *p* < 0.01, *Prmt1* Ctrl vs. *Prmt1* OE: *F_(3;130)_* = 11.41, *p* < 0.01). Data are represented as mean ± SEM; ns, not significant.

We next investigated the necessity of PRMT1 downregulation in developing peripheral pain hypersensitivity. We overexpressed PRMT1 in the ACC using AAV8-P_CMV_-bGlobin-*Prmt1*-P2A-eGFP (Figures 5A,B) and did not detect any remarkable changes in PWTs (Figure 5C) on day 21 after virus expression. We further ligated CPN and observed that, unlike mice with the *Prmt1* Ctrl virus, CPN ligation did not decrease the PWTs (Figure 5C) in mice with the *Prmt1* OE virus. Furthermore, the CFA injection did not change the PWTs (Figure 5D) in mice with the *Prmt1* OE virus. The overexpression of PRMT1 did not notably affect motor function because neither the center time nor travel distance in the open-field test exhibited apparent change (data not shown). Taken together, these results suggest that overexpression of PRMT1 in the ACC prevents the development of peripheral hypersensitivity induced by peripheral inflammation or nerve injury.

## 3. Discussion

In the current study, we examined the possible role of PRMT1 in the development of peripheral pain hypersensitivity. We observed the downregulation of PRMT1 after peripheral nerve injury. Behaviorally, the downregulation of PRMT1 in the ACC was sufficient and necessary to develop peripheral pain hypersensitivity. However, in the *Fmr1* KO mice, the role of the downregulation of PRMT1 on pain perception was abolished. Thus, to our knowledge, this is the first study to show that the downregulation of PRMT1 is essential for peripheral hypersensitivity.

### 3.1. PRMT1 is involved in the peripheral pain hypersensitivity

In this work, we observed the downregulation of PRMT1 in the ACC of a kind of neuropathic pain model with CPN ligation. Also, arginine methylation is an essential part of protein post-translational modification, and PRMT1 is the predominant protein arginine methyltransferases in mammalian cells (Blanc and Richard, 2017). Mo and co-workers reported that the expression of coactivator-associated arginine methyltransferase 1 in the dorsal root ganglia was upregulated after nerve injury, contributing to peripheral hypersensitivity *via* epigenetic regulation (Mo et al., 2018). Unlike Mo′s study, we showed that PRMT1 is involved in pain regulation *via* the activities on FMRP.

Plus, we observed significant pain hypersensitivity through the ethological tests after either PRMT1 knockdown or PRMT1 activity inhibiting. We employed the genetic approach, downregulated the PRMT1 *via* shRNA in the ACC, and observed the apparent peripheral pain hypersensitivity. Thus, we provided solid evidence to show that the activities-dependent downregulation of PRMT1 was sufficient to develop peripheral pain hypersensitivity. Furthermore, overexpression of PRMT1 in ACC rescued mechanical allodynia of mice with peripheral nerve injury; this suggests that the downregulation of PRMT1 was necessary for hypersensitivity. Therefore, we provided new evidence for the protein arginine methylation’s involvement in the CNS’s functions.

### 3.2. Arginine methylation regulates the bound ability of FMRP

FMRP is a crucial factor for the hypersensitivity induced by PRMT1 downregulation. Downregulation of cingulate PRMT1 failed to induce allodynia when FMRP was absent. The FMRP is broadly distributed in the CNS, and the lack of FMRP in other areas may mask PRMT1 downregulation in the ACC. Here we found that knockdown cingulate FMRP prevented the decrease of PWTs induced by nerve injury. Furthermore, nerve injury decreased the PWTs of *Fmr1* KO mice with WT FMRP re-expression in the ACC. These data suggest that the cingulate FMRP is necessary for developing hypersensitivity after nerve injury. Furthermore, deficits of FMRP did not change the expression of PRMT1. Taking together, we concluded that in the ACC, the downregulation of PRMT1 was involved in developing hypersensitivity *via* changing the level of FMRP.

Arginine methylation on the C terminal is crucial to regulating FMRP functionally. Arginine methylation of FMRP was first observed by HeLa cell extracts (Liu and Dreyfuss, 1995). Later, it was reported that a synthetic peptide with RGG sequence presented in human FMRP could be methylated by rat brain extract (Ai et al., 1999). The exact methylated sites were identified in 2006 (Stetler et al., 2006). By mutating arginine to lysine or blocking methylation with adenosine-2′, 3′-dialdehyde (AdOx), it was found that the methylation of the RGG box modulated the quantity of bound mRNA by FMRP (Blackwell et al., 2010). Given that the arginine methylation regulates the condensation of FMRP with the binding RNA (Tsang et al., 2019), we also observed a higher ratio of condensed FMRP in the cultured neurons with the AMI-1 application; the downregulation of PRMT1 may modulate the condensation of FMRP, therefore changing the expression of excitatory synaptic transmission in the ACC.

Meanwhile, our study also provides promising practical pain management to administer drugs to ACC directly. Combined with our previous study (Wang et al., 2017), cannular microinjection delivered an analgesic to ACC, and the PWTs of mice were markedly increased. Therefore, precisely applying analgesics to ACC is a potential option for treating neuropathic pain.

## Data availability statement

The original contributions presented in the study are included in the article/supplementary material, further inquiries can be directed to the corresponding author.

## Ethics statement

The animal study was reviewed and approved by the animal care and use committee of Zhejiang University approved all mice protocols.

## Author contributions

CW, H-FS, and Y-JW performed western blot, immunostaining, mechanical allodynia testing, data analysis, and approved the draft. J-HW, Z-XZ, Y-NL, CW, and LL performed virus injection and allodynia testing, and analyzed the data. CZ provided the Fmr1 KO mice and analyzed data. X-YL designed the experiments, approved the draft, and wrote the paper. All authors contributed to the article and approved the submitted version.

## Funding

This study was supported by the Hangzhou Science and Technology Plan Project (20190101A10), the National Natural Science Foundation of China (31871062, 82071181, and 32271042), the Fundamental Research Funds for the Central Universities, the MOE Frontier Science Center for Brain Science & Brain-Machine Integration, Zhejiang University.

## Conflict of interest

The authors declare that the research was conducted in the absence of any commercial or financial relationships that could be construed as a potential conflict of interest.

## Publisher’s note

All claims expressed in this article are solely those of the authors and do not necessarily represent those of their affiliated organizations, or those of the publisher, the editors and the reviewers. Any product that may be evaluated in this article, or claim that may be made by its manufacturer, is not guaranteed or endorsed by the publisher.
